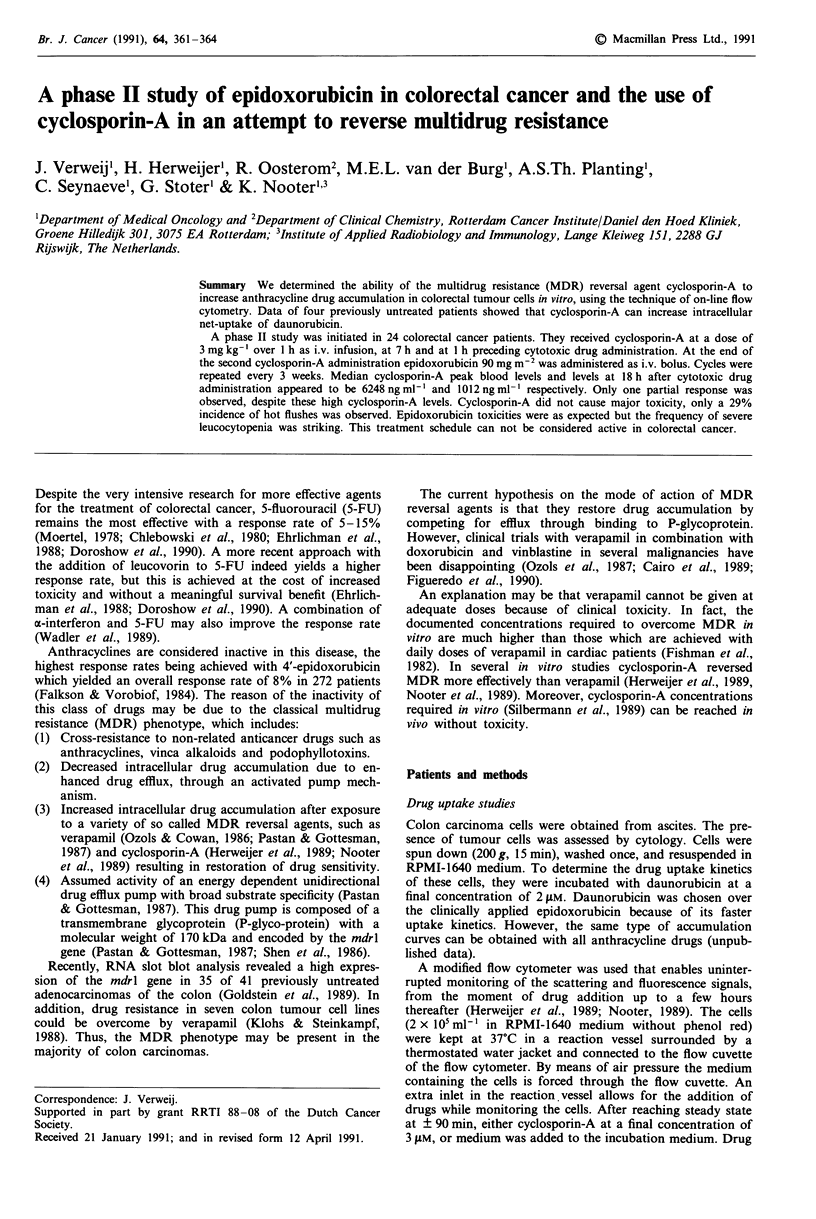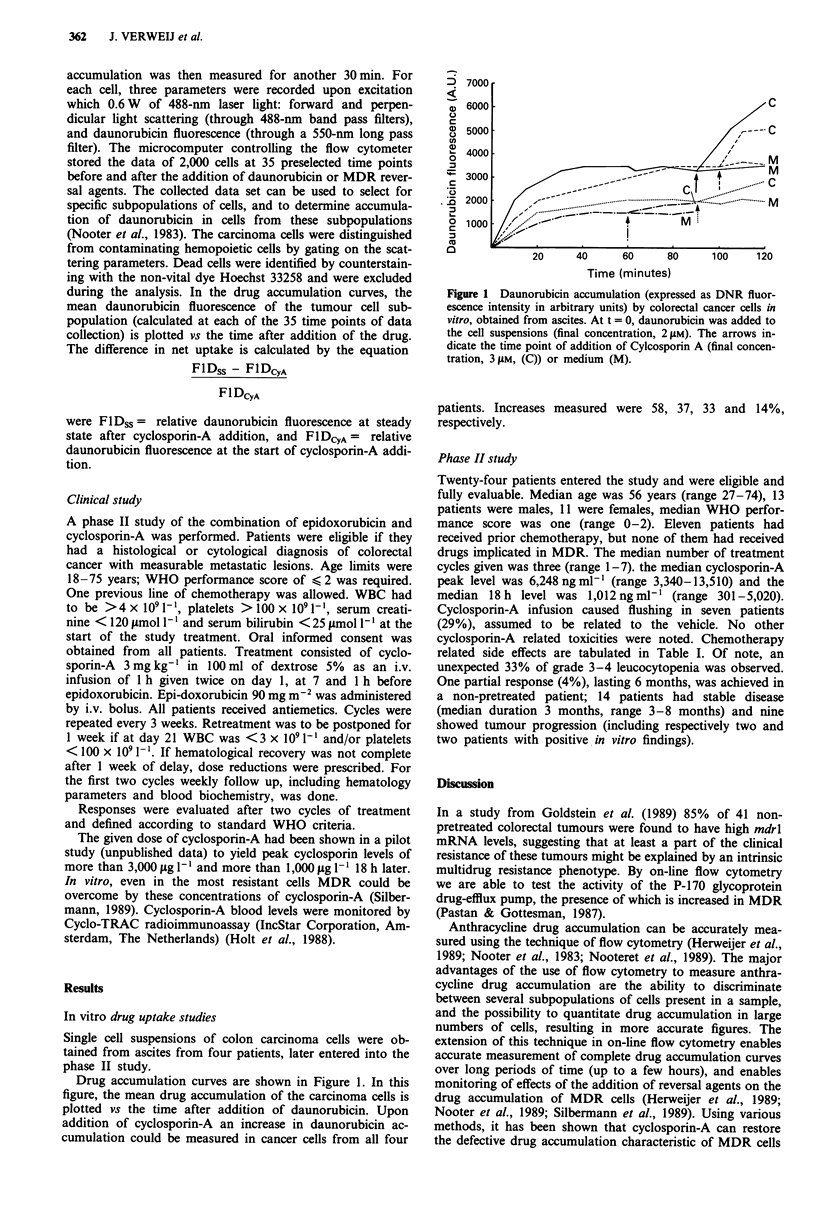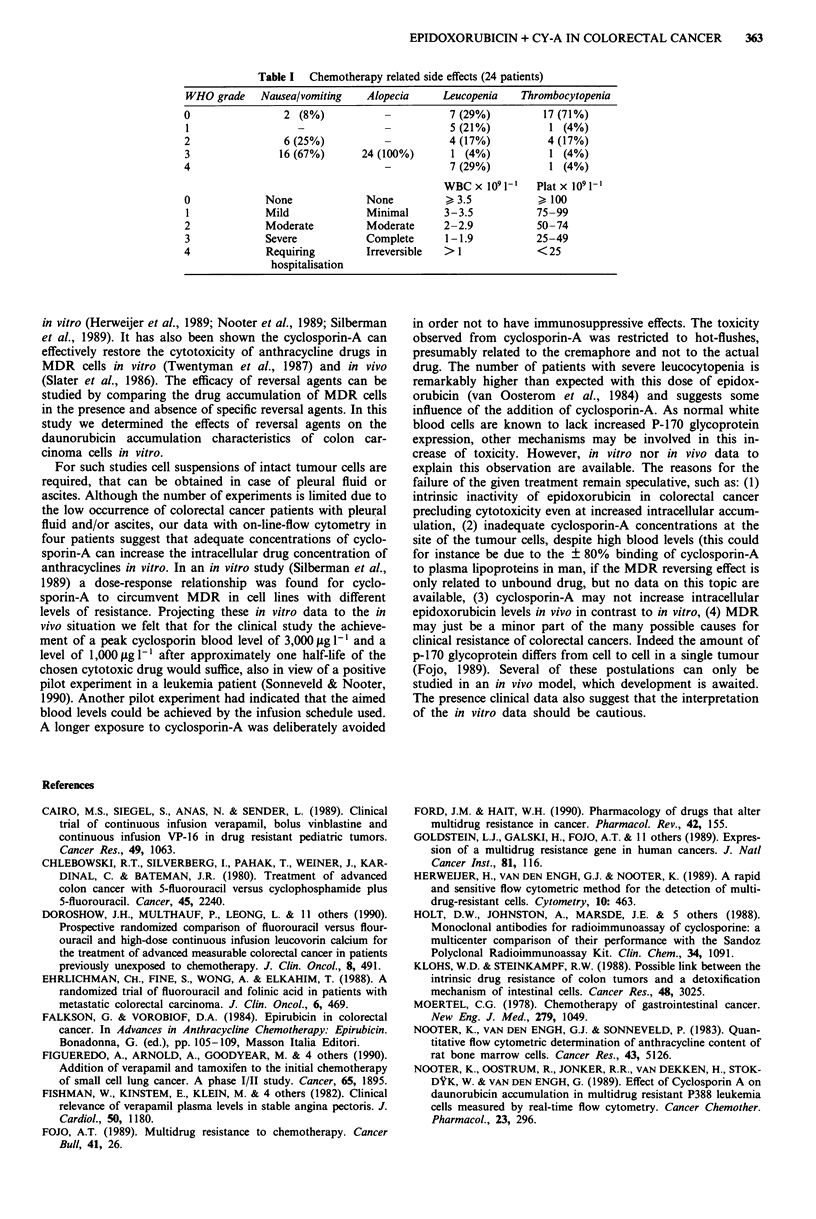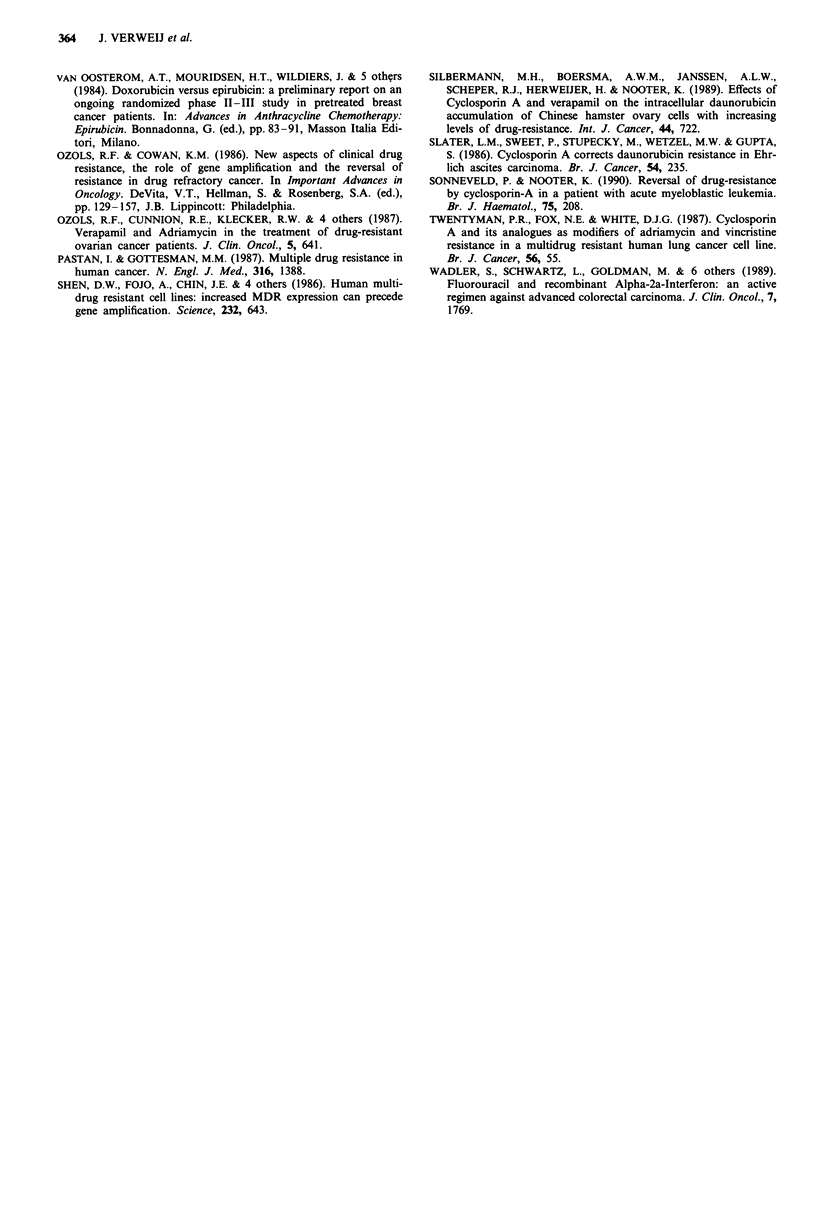# A phase II study of epidoxorubicin in colorectal cancer and the use of cyclosporin-A in an attempt to reverse multidrug resistance.

**DOI:** 10.1038/bjc.1991.307

**Published:** 1991-08

**Authors:** J. Verweij, H. Herweijer, R. Oosterom, M. E. van der Burg, A. S. Planting, C. Seynaeve, G. Stoter, K. Nooter

**Affiliations:** Department of Medical Oncology, Rotterdam Cancer Institute/Daniel den Hoed Kliniek, The Netherlands.

## Abstract

We determined the ability of the multidrug resistance (MDR) reversal agent cyclosporin-A to increase anthracycline drug accumulation in colorectal tumour cells in vitro, using the technique of on-line flow cytometry. Data of four previously untreated patients showed that cyclosporin-A can increase intracellular net-uptake of daunorubicin. A phase II study was initiated in 24 colorectal cancer patients. They received cyclosporin-A at a dose of 3 mg kg-1 over 1 h as i.v. infusion, at 7 h and at 1 h preceding cytotoxic drug administration. At the end of the second cyclosporin-A administration epidoxorubicin 90 mg m-2 was administered as i.v. bolus. Cycles were repeated every 3 weeks. Median cyclosporin-A peak blood levels and levels at 18 h after cytotoxic drug administration appeared to be 6248 ng ml-1 and 1012 ng ml-1 respectively. Only one partial response was observed, despite these high cyclosporin-A levels. Cyclosporin-A did not cause major toxicity, only a 29% incidence of hot flushes was observed. Epidoxorubicin toxicities were as expected but the frequency of severe leucocytopenia was striking. This treatment schedule can not be considered active in colorectal cancer.


Br. J. Cancer (1991), 64, 361–364

© Macmillan Press Ltd., 1991

### A phase II study of epidoxorubicin in colorectal cancer and the use of cyclosporin-A in an attempt to reverse multidrug resistance

J. Verweij[1], H. Herweijer[1], R. Oosterom[2], M.E.L. van der Burg[1], A.S.Th. Planting[1], C. Seynaeve[1], G. Stoter[1] & K. Nooter[1,3]

[1]*Department of Medical Oncology and* [2]*Department of Clinical Chemistry, Rotterdam Cancer Institute/Daniel den Hoed Kliniek, Groene Hilledijk 301, 3075 EA Rotterdam;* [3]*Institute of Applied Radiobiology and Immunology, Lange Kleiweg 151, 2288 GJ Rijswijk, The Netherlands.*

**Summary** We determined the ability of the multidrug resistance (MDR) reversal agent cyclosporin-A to increase anthracycline drug accumulation in colorectal tumour cells *in vitro*, using the technique of on-line flow cytometry. Data of four previously untreated patients showed that cyclosporin-A can increase intracellular net-uptake of daunorubicin.

A phase II study was initiated in 24 colorectal cancer patients. They received cyclosporin-A at a dose of 3 mg kg$^{-1}$ over 1 h as i.v. infusion, at 7 h and at 1 h preceding cytotoxic drug administration. At the end of the second cyclosporin-A administration epidoxorubicin 90 mg m$^{-2}$ was administered as i.v. bolus. Cycles were repeated every 3 weeks. Median cyclosporin-A peak blood levels and levels at 18 h after cytotoxic drug administration appeared to be 6248 ng ml$^{-1}$ and 1012 ng ml$^{-1}$ respectively. Only one partial response was observed, despite these high cyclosporin-A levels. Cyclosporin-A did not cause major toxicity, only a 29% incidence of hot flushes was observed. Epidoxorubicin toxicities were as expected but the frequency of severe leucocytopenia was striking. This treatment schedule can not be considered active in colorectal cancer.

Despite the very intensive research for more effective agents for the treatment of colorectal cancer, 5-fluorouracil (5-FU) remains the most effective with a response rate of 5–15% (Moertel, 1978; Chlebowski *et al.*, 1980; Ehrlichman *et al.*, 1988; Doroshow *et al.*, 1990). A more recent approach with the addition of leucovorin to 5-FU indeed yields a higher response rate, but this is achieved at the cost of increased toxicity and without a meaningful survival benefit (Ehrlichman *et al.*, 1988; Doroshow *et al.*, 1990). A combination of α-interferon and 5-FU may also improve the response rate (Wadler *et al.*, 1989).

Anthracyclines are considered inactive in this disease, the highest response rates being achieved with 4′-epidoxorubicin which yielded an overall response rate of 8% in 272 patients (Falkson & Vorobiof, 1984). The reason of the inactivity of this class of drugs may be due to the classical multidrug resistance (MDR) phenotype, which includes:

(1) Cross-resistance to non-related anticancer drugs such as anthracyclines, vinca alkaloids and podophyllotoxins.
(2) Decreased intracellular drug accumulation due to enhanced drug efflux, through an activated pump mechanism.
(3) Increased intracellular drug accumulation after exposure to a variety of so called MDR reversal agents, such as verapamil (Ozols & Cowan, 1986; Pastan & Gottesman, 1987) and cyclosporin-A (Herweijer *et al.*, 1989; Nooter *et al.*, 1989) resulting in restoration of drug sensitivity.
(4) Assumed activity of an energy dependent unidirectional drug efflux pump with broad substrate specificity (Pastan & Gottesman, 1987). This drug pump is composed of a transmembrane glycoprotein (P-glyco-protein) with a molecular weight of 170 kDa and encoded by the *mdr*1 gene (Pastan & Gottesman, 1987; Shen *et al.*, 1986).

Recently, RNA slot blot analysis revealed a high expression of the *mdr*1 gene in 35 of 41 previously untreated adenocarcinomas of the colon (Goldstein *et al.*, 1989). In addition, drug resistance in seven colon tumour cell lines could be overcome by verapamil (Klohs & Steinkampf, 1988). Thus, the MDR phenotype may be present in the majority of colon carcinomas.

Correspondence: J. Verweij.
Supported in part by grant RRTI 88-08 of the Dutch Cancer Society.
Received 21 January 1991; and in revised form 12 April 1991.

The current hypothesis on the mode of action of MDR reversal agents is that they restore drug accumulation by competing for efflux through binding to P-glycoprotein. However, clinical trials with verapamil in combination with doxorubicin and vinblastine in several malignancies have been disappointing (Ozols *et al.*, 1987; Cairo *et al.*, 1989; Figueredo *et al.*, 1990).

An explanation may be that verapamil cannot be given at adequate doses because of clinical toxicity. In fact, the documented concentrations required to overcome MDR *in vitro* are much higher than those which are achieved with daily doses of verapamil in cardiac patients (Fishman *et al.*, 1982). In several *in vitro* studies cyclosporin-A reversed MDR more effectively than verapamil (Herweijer *et al.*, 1989, Nooter *et al.*, 1989). Moreover, cyclosporin-A concentrations required *in vitro* (Silbermann *et al.*, 1989) can be reached *in vivo* without toxicity.

### Patients and methods

### *Drug uptake studies*

Colon carcinoma cells were obtained from ascites. The presence of tumour cells was assessed by cytology. Cells were spun down (200 *g*, 15 min), washed once, and resuspended in RPMI-1640 medium. To determine the drug uptake kinetics of these cells, they were incubated with daunorubicin at a final concentration of 2 μM. Daunorubicin was chosen over the clinically applied epidoxorubicin because of its faster uptake kinetics. However, the same type of accumulation curves can be obtained with all anthracycline drugs (unpublished data).

A modified flow cytometer was used that enables uninterrupted monitoring of the scattering and fluorescence signals, from the moment of drug addition up to a few hours thereafter (Herweijer *et al.*, 1989; Nooter, 1989). The cells ($2 \times 10^5$ ml$^{-1}$ in RPMI-1640 medium without phenol red) were kept at 37°C in a reaction vessel surrounded by a thermostated water jacket and connected to the flow cuvette of the flow cytometer. By means of air pressure the medium containing the cells is forced through the flow cuvette. An extra inlet in the reaction vessel allows for the addition of drugs while monitoring the cells. After reaching steady state at ± 90 min, either cyclosporin-A at a final concentration of 3 μM, or medium was added to the incubation medium. Drug accumulation was then measured for another 30 min. For each cell, three parameters were recorded upon excitation which 0.6 W of 488-nm laser light: forward and perpendicular light scattering (through 488-nm band pass filters), and daunorubicin fluorescence (through a 550-nm long pass filter). The microcomputer controlling the flow cytometer stored the data of 2,000 cells at 35 preselected time points before and after the addition of daunorubicin or MDR reversal agents. The collected data set can be used to select for specific subpopulations of cells, and to determine accumulation of daunorubicin in cells from these subpopulations (Nooter *et al.*, 1983). The carcinoma cells were distinguished from contaminating hemopoietic cells by gating on the scattering parameters. Dead cells were identified by counterstaining with the non-vital dye Hoechst 33258 and were excluded during the analysis. In the drug accumulation curves, the mean daunorubicin fluorescence of the tumour cell subpopulation (calculated at each of the 35 time points of data collection) is plotted *vs* the time after addition of the drug. The difference in net uptake is calculated by the equation

$$\frac{FlD_{SS} - FlD_{CyA}}{FlD_{CyA}}$$

were $FlD_{SS}$ = relative daunorubicin fluorescence at steady state after cyclosporin-A addition, and $FlD_{CyA}$ = relative daunorubicin fluorescence at the start of cyclosporin-A addition.

### *Clinical study*

A phase II study of the combination of epidoxorubicin and cyclosporin-A was performed. Patients were eligible if they had a histological or cytological diagnosis of colorectal cancer with measurable metastatic lesions. Age limits were 18–75 years; WHO performance score of $\leqslant 2$ was required. One previous line of chemotherapy was allowed. WBC had to be $> 4 \times 10^9\, l^{-1}$, platelets $> 100 \times 10^9\, l^{-1}$, serum creatinine $< 120\, \mu mol\, l^{-1}$ and serum bilirubin $< 25\, \mu mol\, l^{-1}$ at the start of the study treatment. Oral informed consent was obtained from all patients. Treatment consisted of cyclosporin-A $3\, mg\, kg^{-1}$ in 100 ml of dextrose 5% as an i.v. infusion of 1 h given twice on day 1, at 7 and 1 h before epidoxorubicin. Epi-doxorubicin $90\, mg\, m^{-2}$ was administered by i.v. bolus. All patients received antiemetics. Cycles were repeated every 3 weeks. Retreatment was to be postponed for 1 week if at day 21 WBC was $< 3 \times 10^9\, l^{-1}$ and/or platelets $< 100 \times 10^9\, l^{-1}$. If hematological recovery was not complete after 1 week of delay, dose reductions were prescribed. For the first two cycles weekly follow up, including hematology parameters and blood biochemistry, was done.

Responses were evaluated after two cycles of treatment and defined according to standard WHO criteria.

The given dose of cyclosporin-A had been shown in a pilot study (unpublished data) to yield peak cyclosporin levels of more than $3{,}000\, \mu g\, l^{-1}$ and more than $1{,}000\, \mu g\, l^{-1}$ 18 h later. *In vitro*, even in the most resistant cells MDR could be overcome by these concentrations of cyclosporin-A (Silbermann, 1989). Cyclosporin-A blood levels were monitored by Cyclo-TRAC radioimmunoassay (IncStar Corporation, Amsterdam, The Netherlands) (Holt *et al.*, 1988).

### Results

### In vitro *drug uptake studies*

Single cell suspensions of colon carcinoma cells were obtained from ascites from four patients, later entered into the phase II study.

Drug accumulation curves are shown in Figure 1. In this figure, the mean drug accumulation of the carcinoma cells is plotted *vs* the time after addition of daunorubicin. Upon addition of cyclosporin-A an increase in daunorubicin accumulation could be measured in cancer cells from all four patients. Increases measured were 58, 37, 33 and 14%, respectively.

**Figure 1** Daunorubicin accumulation (expressed as DNR fluorescence intensity in arbitrary units) by colorectal cancer cells *in vitro*, obtained from ascites. At t = 0, daunorubicin was added to the cell suspensions (final concentration, 2 $\mu$M). The arrows indicate the time point of addition of Cylcosporin A (final concentration, 3 $\mu$M, (C)) or medium (M).

### *Phase II study*

Twenty-four patients entered the study and were eligible and fully evaluable. Median age was 56 years (range 27–74), 13 patients were males, 11 were females, median WHO performance score was one (range 0–2). Eleven patients had received prior chemotherapy, but none of them had received drugs implicated in MDR. The median number of treatment cycles given was three (range 1–7). the median cyclosporin-A peak level was $6{,}248\, ng\, ml^{-1}$ (range 3,340–13,510) and the median 18 h level was $1{,}012\, ng\, ml^{-1}$ (range 301–5,020). Cyclosporin-A infusion caused flushing in seven patients (29%), assumed to be related to the vehicle. No other cyclosporin-A related toxicities were noted. Chemotherapy related side effects are tabulated in Table I. Of note, an unexpected 33% of grade 3–4 leucocytopenia was observed. One partial response (4%), lasting 6 months, was achieved in a non-pretreated patient; 14 patients had stable disease (median duration 3 months, range 3–8 months) and nine showed tumour progression (including respectively two and two patients with positive *in vitro* findings).

### Discussion

In a study from Goldstein *et al.* (1989) 85% of 41 non-pretreated colorectal tumours were found to have high *mdr*1 mRNA levels, suggesting that at least a part of the clinical resistance of these tumours might be explained by an intrinsic multidrug resistance phenotype. By on-line flow cytometry we are able to test the activity of the P-170 glycoprotein drug-efflux pump, the presence of which is increased in MDR (Pastan & Gottesman, 1987).

Anthracycline drug accumulation can be accurately measured using the technique of flow cytometry (Herweijer *et al.*, 1989; Nooter *et al.*, 1983; Nooteret *et al.*, 1989). The major advantages of the use of flow cytometry to measure anthracycline drug accumulation are the ability to discriminate between several subpopulations of cells present in a sample, and the possibility to quantitate drug accumulation in large numbers of cells, resulting in more accurate figures. The extension of this technique in on-line flow cytometry enables accurate measurement of complete drug accumulation curves over long periods of time (up to a few hours), and enables monitoring of effects of the addition of reversal agents on the drug accumulation of MDR cells (Herweijer *et al.*, 1989; Nooter *et al.*, 1989; Silbermann *et al.*, 1989). Using various methods, it has been shown that cyclosporin-A can restore the defective drug accumulation characteristic of MDR cells

**Table I** Chemotherapy related side effects (24 patients)

| *WHO grade* | *Nausea/vomiting* | *Alopecia* | *Leucopenia* | *Thrombocytopenia* |
|---|---|---|---|---|
| 0 | 2 (8%) | – | 7 (29%) | 17 (71%) |
| 1 | – | – | 5 (21%) | 1 (4%) |
| 2 | 6 (25%) | – | 4 (17%) | 4 (17%) |
| 3 | 16 (67%) | 24 (100%) | 1 (4%) | 1 (4%) |
| 4 | | – | 7 (29%) | 1 (4%) |
| | | | WBC × $10^9\,l^{-1}$ | Plat × $10^9\,l^{-1}$ |
| 0 | None | None | ⩾3.5 | ⩾100 |
| 1 | Mild | Minimal | 3–3.5 | 75–99 |
| 2 | Moderate | Moderate | 2–2.9 | 50–74 |
| 3 | Severe | Complete | 1–1.9 | 25–49 |
| 4 | Requiring hospitalisation | Irreversible | >1 | <25 |

*in vitro* (Herweijer *et al.*, 1989; Nooter *et al.*, 1989; Silberman *et al.*, 1989). It has also been shown the cyclosporin-A can effectively restore the cytotoxicity of anthracycline drugs in MDR cells *in vitro* (Twentyman *et al.*, 1987) and *in vivo* (Slater *et al.*, 1986). The efficacy of reversal agents can be studied by comparing the drug accumulation of MDR cells in the presence and absence of specific reversal agents. In this study we determined the effects of reversal agents on the daunorubicin accumulation characteristics of colon carcinoma cells *in vitro*.

For such studies cell suspensions of intact tumour cells are required, that can be obtained in case of pleural fluid or ascites. Although the number of experiments is limited due to the low occurrence of colorectal cancer patients with pleural fluid and/or ascites, our data with on-line-flow cytometry in four patients suggest that adequate concentrations of cyclosporin-A can increase the intracellular drug concentration of anthracyclines *in vitro*. In an *in vitro* study (Silberman *et al.*, 1989) a dose-response relationship was found for cyclosporin-A to circumvent MDR in cell lines with different levels of resistance. Projecting these *in vitro* data to the *in vivo* situation we felt that for the clinical study the achievement of a peak cyclosporin blood level of 3,000 $\mu g\,l^{-1}$ and a level of 1,000 $\mu g\,l^{-1}$ after approximately one half-life of the chosen cytotoxic drug would suffice, also in view of a positive pilot experiment in a leukemia patient (Sonneveld & Nooter, 1990). Another pilot experiment had indicated that the aimed blood levels could be achieved by the infusion schedule used. A longer exposure to cyclosporin-A was deliberately avoided in order not to have immunosuppressive effects. The toxicity observed from cyclosporin-A was restricted to hot-flushes, presumably related to the cremaphore and not to the actual drug. The number of patients with severe leucocytopenia is remarkably higher than expected with this dose of epidoxorubicin (van Oosterom *et al.*, 1984) and suggests some influence of the addition of cyclosporin-A. As normal white blood cells are known to lack increased P-170 glycoprotein expression, other mechanisms may be involved in this increase of toxicity. However, *in vitro* nor *in vivo* data to explain this observation are available. The reasons for the failure of the given treatment remain speculative, such as: (1) intrinsic inactivity of epidoxorubicin in colorectal cancer precluding cytotoxicity even at increased intracellular accumulation, (2) inadequate cyclosporin-A concentrations at the site of the tumour cells, despite high blood levels (this could for instance be due to the ± 80% binding of cyclosporin-A to plasma lipoproteins in man, if the MDR reversing effect is only related to unbound drug, but no data on this topic are available, (3) cyclosporin-A may not increase intracellular epidoxorubicin levels *in vivo* in contrast to *in vitro*, (4) MDR may just be a minor part of the many possible causes for clinical resistance of colorectal cancers. Indeed the amount of p-170 glycoprotein differs from cell to cell in a single tumour (Fojo, 1989). Several of these postulations can only be studied in an *in vivo* model, which development is awaited. The presence clinical data also suggest that the interpretation of the *in vitro* data should be cautious.

### References

CAIRO, M.S., SIEGEL, S., ANAS, N. & SENDER, L. (1989). Clinical trial of continuous infusion verapamil, bolus vinblastine and continuous infusion VP-16 in drug resistant pediatric tumors. *Cancer Res.*, **49**, 1063.

CHLEBOWSKI, R.T., SILVERBERG, I., PAHAK, T., WEINER, J., KARDINAL, C. & BATEMAN, J.R. (1980). Treatment of advanced colon cancer with 5-fluorouracil versus cyclophosphamide plus 5-fluorouracil. *Cancer*, **45**, 2240.

DOROSHOW, J.H., MULTHAUF, P., LEONG, L. & 11 others (1990). Prospective randomized comparison of fluorouracil versus flourouracil and high-dose continuous infusion leucovorin calcium for the treatment of advanced measurable colorectal cancer in patients previously unexposed to chemotherapy. *J. Clin. Oncol.*, **8**, 491.

EHRLICHMAN, CH., FINE, S., WONG, A. & ELKAHIM, T. (1988). A randomized trial of fluorouracil and folinic acid in patients with metastatic colorectal carcinoma. *J. Clin. Oncol.*, **6**, 469.

FALKSON, G. & VOROBIOF, D.A. (1984). Epirubicin in colorectal cancer. In *Advances in Anthracycline Chemotherapy: Epirubicin*. Bonadonna, G. (ed.), pp. 105–109, Masson Italia Editori.

FIGUEREDO, A., ARNOLD, A., GOODYEAR, M. & 4 others (1990). Addition of verapamil and tamoxifen to the initial chemotherapy of small cell lung cancer. A phase I/II study. *Cancer*, **65**, 1895.

FISHMAN, W., KINSTEM, E., KLEIN, M. & 4 others (1982). Clinical relevance of verapamil plasma levels in stable angina pectoris. *J. Cardiol.*, **50**, 1180.

FOJO, A.T. (1989). Multidrug resistance to chemotherapy. *Cancer Bull*, **41**, 26.

FORD, J.M. & HAIT, W.H. (1990). Pharmacology of drugs that alter multidrug resistance in cancer. *Pharmacol. Rev.*, **42**, 155.

GOLDSTEIN, L.J., GALSKI, H., FOJO, A.T. & 11 others (1989). Expression of a multidrug resistance gene in human cancers. *J. Natl Cancer Inst.*, **81**, 116.

HERWEIJER, H., VAN DEN ENGH, G.J. & NOOTER, K. (1989). A rapid and sensitive flow cytometric method for the detection of multidrug-resistant cells. *Cytometry*, **10**: 463.

HOLT, D.W., JOHNSTON, A., MARSDE, J.E. & 5 others (1988). Monoclonal antibodies for radioimmunoassay of cyclosporine: a multicenter comparison of their performance with the Sandoz Polyclonal Radioimmunoassay Kit. *Clin. Chem.*, **34**, 1091.

KLOHS, W.D. & STEINKAMPF, R.W. (1988). Possible link between the intrinsic drug resistance of colon tumors and a detoxification mechanism of intestinal cells. *Cancer Res.*, **48**, 3025.

MOERTEL, C.G. (1978). Chemotherapy of gastrointestinal cancer. *New Eng. J. Med.*, **279**, 1049.

NOOTER, K., VAN DEN ENGH, G.J. & SONNEVELD, P. (1983). Quantitative flow cytometric determination of anthracycline content of rat bone marrow cells. *Cancer Res.*, **43**, 5126.

NOOTER, K., OOSTRUM, R., JONKER, R.R., VAN DEKKEN, H., STOKDŸK, W. & VAN DEN ENGH, G. (1989). Effect of Cyclosporin A on daunorubicin accumulation in multidrug resistant P388 leukemia cells measured by real-time flow cytometry. *Cancer Chemother. Pharmacol.*, **23**, 296.

VAN OOSTEROM, A.T., MOURIDSEN, H.T., WILDIERS, J. & 5 others (1984). Doxorubicin versus epirubicin: a preliminary report on an ongoing randomized phase II-III study in pretreated breast cancer patients. In: *Advances in Anthracycline Chemotherapy: Epirubicin*. Bonnadonna, G. (ed.), pp. 83-91, Masson Italia Editori, Milano.

OZOLS, R.F. & COWAN, K.M. (1986). New aspects of clinical drug resistance, the role of gene amplification and the reversal of resistance in drug refractory cancer. In *Important Advances in Oncology*. DeVita, V.T., Hellman, S. & Rosenberg, S.A. (ed.), pp. 129-157, J.B. Lippincott: Philadelphia.

OZOLS, R.F., CUNNION, R.E., KLECKER, R.W. & 4 others (1987). Verapamil and Adriamycin in the treatment of drug-resistant ovarian cancer patients. *J. Clin. Oncol.*, **5**, 641.

PASTAN, I. & GOTTESMAN, M.M. (1987). Multiple drug resistance in human cancer. *N. Engl. J. Med.*, **316**, 1388.

SHEN, D.W., FOJO, A., CHIN, J.E. & 4 others (1986). Human multidrug resistant cell lines: increased MDR expression can precede gene amplification. *Science*, **232**, 643.

SILBERMANN, M.H., BOERSMA, A.W.M., JANSSEN, A.L.W., SCHEPER, R.J., HERWEIJER, H. & NOOTER, K. (1989). Effects of Cyclosporin A and verapamil on the intracellular daunorubicin accumulation of Chinese hamster ovary cells with increasing levels of drug-resistance. *Int. J. Cancer*, **44**, 722.

SLATER, L.M., SWEET, P., STUPECKY, M., WETZEL, M.W. & GUPTA, S. (1986). Cyclosporin A corrects daunorubicin resistance in Ehrlich ascites carcinoma. *Br. J. Cancer*, **54**, 235.

SONNEVELD, P. & NOOTER, K. (1990). Reversal of drug-resistance by cyclosporin-A in a patient with acute myeloblastic leukemia. *Br. J. Haematol.*, **75**, 208.

TWENTYMAN, P.R., FOX, N.E. & WHITE, D.J.G. (1987). Cyclosporin A and its analogues as modifiers of adriamycin and vincristine resistance in a multidrug resistant human lung cancer cell line. *Br. J. Cancer*, **56**, 55.

WADLER, S., SCHWARTZ, L., GOLDMAN, M. & 6 others (1989). Fluorouracil and recombinant Alpha-2a-Interferon: an active regimen against advanced colorectal carcinoma. *J. Clin. Oncol.*, **7**, 1769.